# Application of multiple-finding segmentation utilizing Mask R-CNN-based deep learning in a rat model of drug-induced liver injury

**DOI:** 10.1038/s41598-023-44897-8

**Published:** 2023-10-16

**Authors:** Eun Bok Baek, Jaeku Lee, Ji-Hee Hwang, Heejin Park, Byoung-Seok Lee, Yong-Bum Kim, Sang-Yeop Jun, Jun Her, Hwa-Young Son, Jae-Woo Cho

**Affiliations:** 1 College of Veterinary Medicine, Chungnam National University, 99 Daehak-ro, Yuseong-gu, Daejeon, 34134 Republic of Korea; 2 Research and Development Team, LAC Inc, Seoul, Republic of Korea; 3 Toxicologic Pathology Research Group, Department of Advanced Toxicology Research, Korea Institute of Toxicology, 141 Gajeong-ro, Yuseong-gu, Daejeon, 34114 Republic of Korea; 4 Department of Advanced Toxicology Research, Korea Institute of Toxicology, Daejeon, 34114 Republic of Korea

**Keywords:** Computational biology and bioinformatics, Gastroenterology

## Abstract

Drug-induced liver injury (DILI) presents significant diagnostic challenges, and recently artificial intelligence-based deep learning technology has been used to predict various hepatic findings. In this study, we trained a set of Mask R-CNN-based deep algorithms to learn and quantify typical toxicant induced-histopathological lesions, portal area, and connective tissue in Sprague Dawley rats. We compared a set of single-finding models (SFMs) and a combined multiple-finding model (MFM) for their ability to simultaneously detect, classify, and quantify multiple hepatic findings on rat liver slide images. All of the SFMs yielded mean average precision (mAP) values above 85%, suggesting that the models had been successfully established. The MFM showed better performance than the SFMs, with a total mAP value of 92.46%. We compared the model predictions for slide images with ground-truth annotations generated by an accredited pathologist. For the MFM, the overall and individual finding predictions were highly correlated with the annotated areas, with R-squared values of 0.852, 0.952, 0.999, 0.990, and 0.958 being obtained for portal area, infiltration, necrosis, vacuolation, and connective tissue (including fibrosis), respectively. Our results indicate that the proposed MFM could be a useful tool for detecting and predicting multiple hepatic findings in basic non-clinical study settings.

## Introduction

Drug-induced liver injury (DILI) is the most frequent safety-related cause of drug withdrawn from the market^1^. The diverse histological findings of DILI can include inflammation, necrosis, cholestasis, fibrosis, nodular regeneration, vascular injury, and bile duct destruction. In a histological sample, these lesions may exist in combinations that can be difficult to discriminate^2,3^. Therefore, pathologists must provide an expert interpretation of the tissue changes in light of the patient’s medical and pharmaceutical history when they evaluate a case of DILI. Because of the potential complexity of these cases, a systematic approach is recommended^4^. Examination of animal models is a critical step in the preclinical investigation of DILI^5^. Several animal models have been studied for predicting DILI, with acetaminophen (APAP) hepatotoxicity having been extensively investigated^6^. Although animal studies are useful, they can only predict about 70% of human hepatotoxicity outcomes^5^.

Digital pathology is a sub-field of pathology that uses tools and systems for digitizing, evaluating, and analyzing pathology slides and associated metadata^7^. Researchers have used deep-learning techniques for both clinical and non-clinical applications, particularly those for which classical imaging analysis methods cannot be automated^8,9^. In recent years, artificial intelligence (AI)-based digital pathology has shown great promise as a means to increase healthcare availability and accuracy in many aspects of medicine^10–12^. In addition to the importance of its growing sub-segments, such as digital molecular pathology and pathology informatics, digital pathology could potentially alter the traditional core functions of pathology and improve the workflow of pathologists^10,13,14^.

Medical imaging research has explored various machine-learning techniques, including many classifier and clustering algorithms^15^. A technique that has shown great promise is the mask region-based convolutional neural network (Mask R-CNN), a detection-based segmentation model^16,17^ comprised of two main stages: (i) object detection and localization; and (ii) using the features of the detected regions to classify them, assign their final localization, and segment them^16^. Recently, Mask R-CNN-based approaches have been used in medical research^17–19^.

Most DILI-related liver lesions are complicated, and recognizing such complex lesions is critical to diagnosing DILI and assessing its progression and/or severity. In the present study, we applied Mask R-CNN-based deep learning to develop a more efficient tool for screening and examining complex liver lesions in a rat model of liver injury. To test the real-world usefulness of our prediction method, we established a set of single-finding models (SFMs) and a multiple-finding model (MFM). We trained, validated, and tested the AI algorithms by comparing the SFM- and MFM-predicted findings with ground-truth annotations generated by an accredited toxicological pathologist.

## Results

### Establishment of the Mask R-CNN algorithms for hepatic lesion and normal feature prediction

The Mask R-CNN was trained to identify various hepatic injury lesions (infiltration, necrosis, fibrosis, vacuolation) and normal features (portal area and connective tissue) to enable the algorithm to discriminate among these findings. To induce liver injury, N-nitrosodimethylamine (NDMA), APAP, or corn oil was applied for the periods listed in Table 1. Each model was established by training it on slide images from the control and drug-treated animals based on a given lesion or normal feature, regardless of treatment. The representative histopathological findings for NDMA-, APAP-, and corn oil-treated animals were fibrosis, necrosis and infiltration, and vacuolation, respectively (Supplementary materials S1 and S2).

**Table 1:** Animal study design for liver sample preparation.

Group	Control	NDMA	APAP	Corn oil
Test material	Distilled water	NDMA	APAP	Corn oil
Dose	10 ml/kg	10 mg/kg	2500 or 1000 mg/kg mg/kg	10 ml/kg
No. of animals (male/female)	6/6	7/7	7/7	7/7
Duration of administration	4 weeks	4 weeks	3 or 7 days	4 weeks
Route of administration	Oral gavage	i.p	Oral gavage	Oral gavage
Dosing regimen	Daily	Three times per week	Single or six times	Daily
No. of slide images	1–25	26–50	51–75	76–100

**Figure 1:**
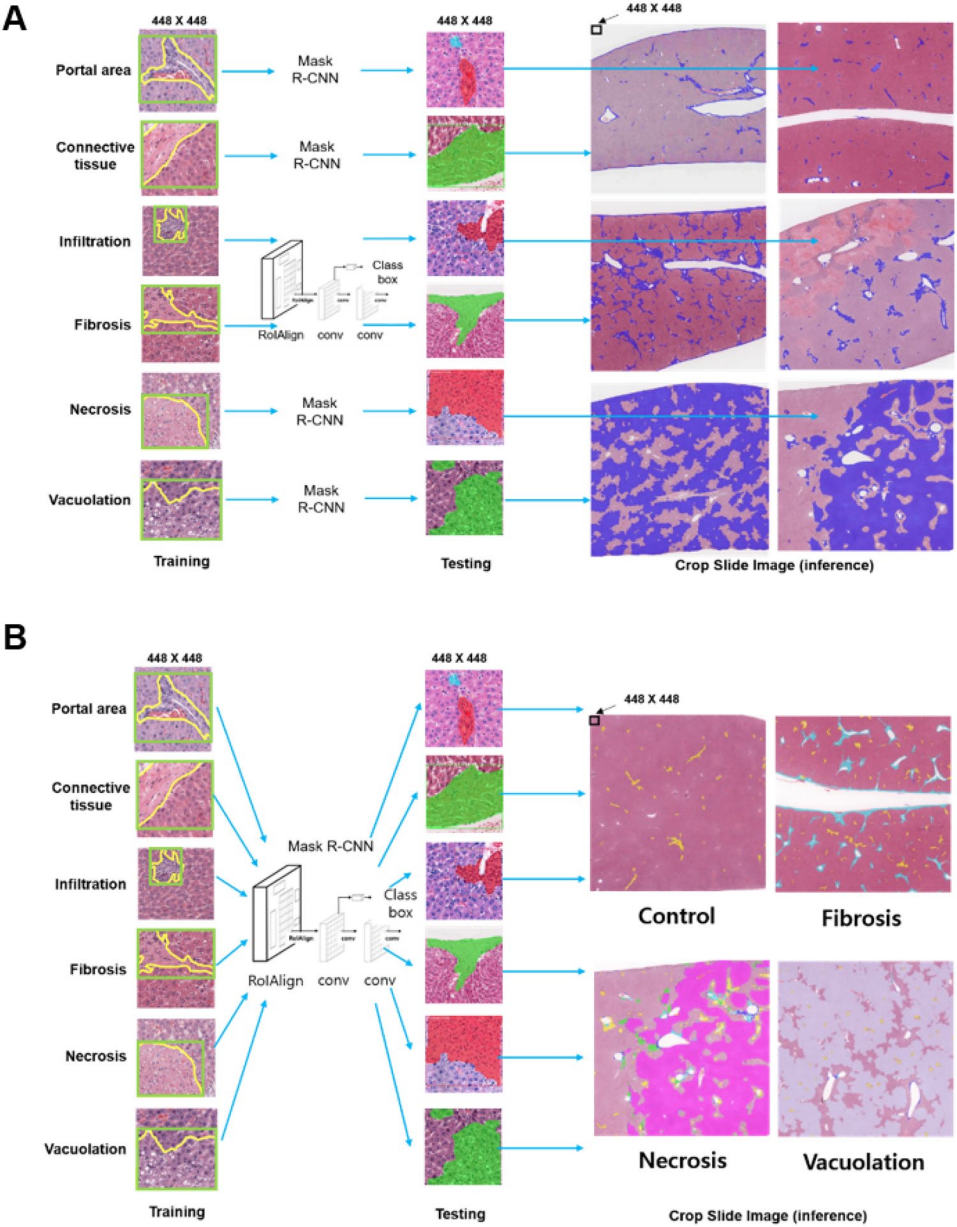
Schematic of Mask R-CNN algorithm procedures for hepatic lesions and normal features in the single-finding models (**A**) and multiple-finding model (**B**).

**Figure 2:**
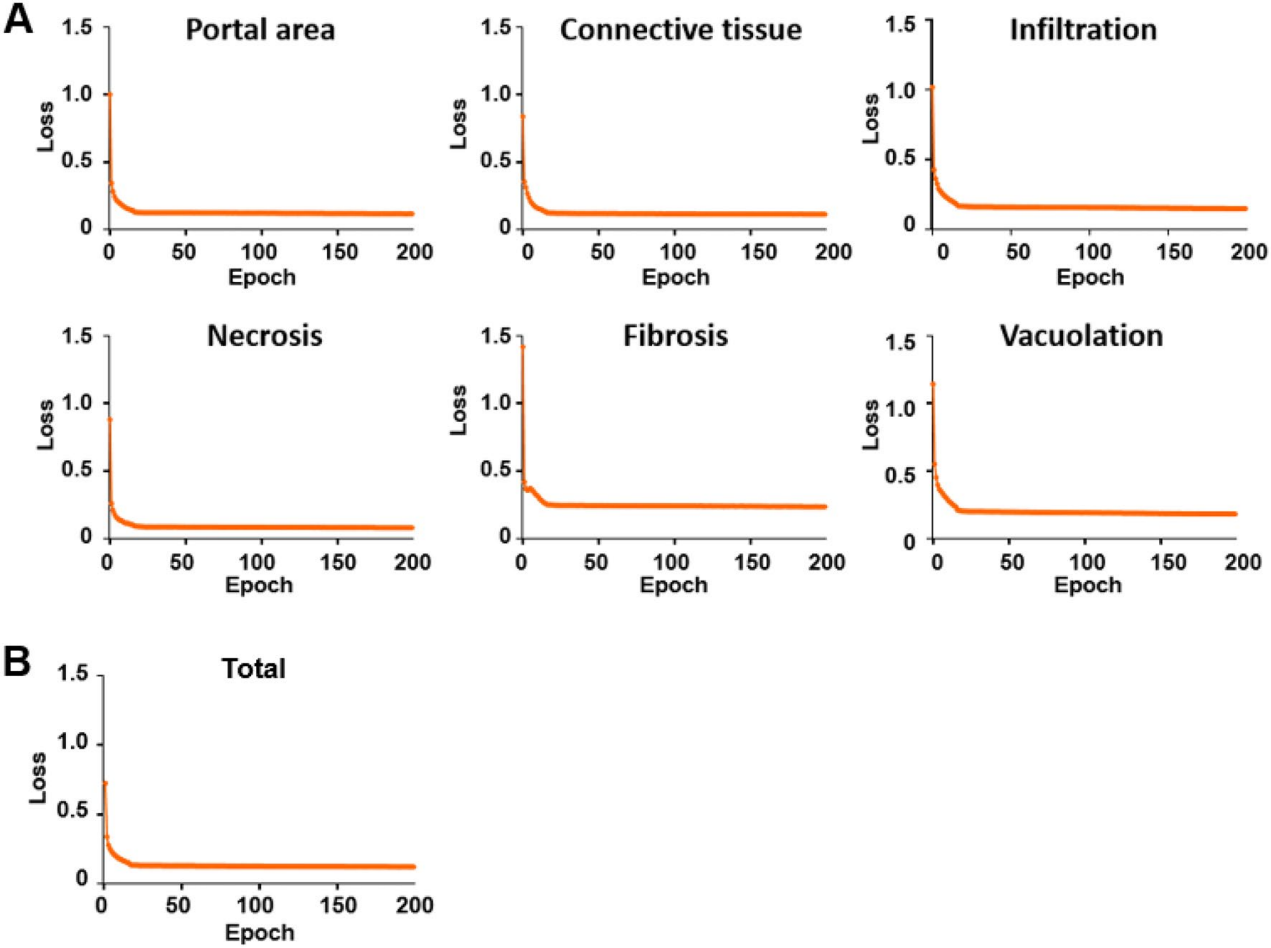
Total training loss during training of single- and multiple-finding models. (**A**) Total loss of portal, connective tissue, infiltration, necrosis, fibrosis, and vacuolation in each single-finding model. (**B**) Total loss in the multiple-finding model.

**Figure 3:**
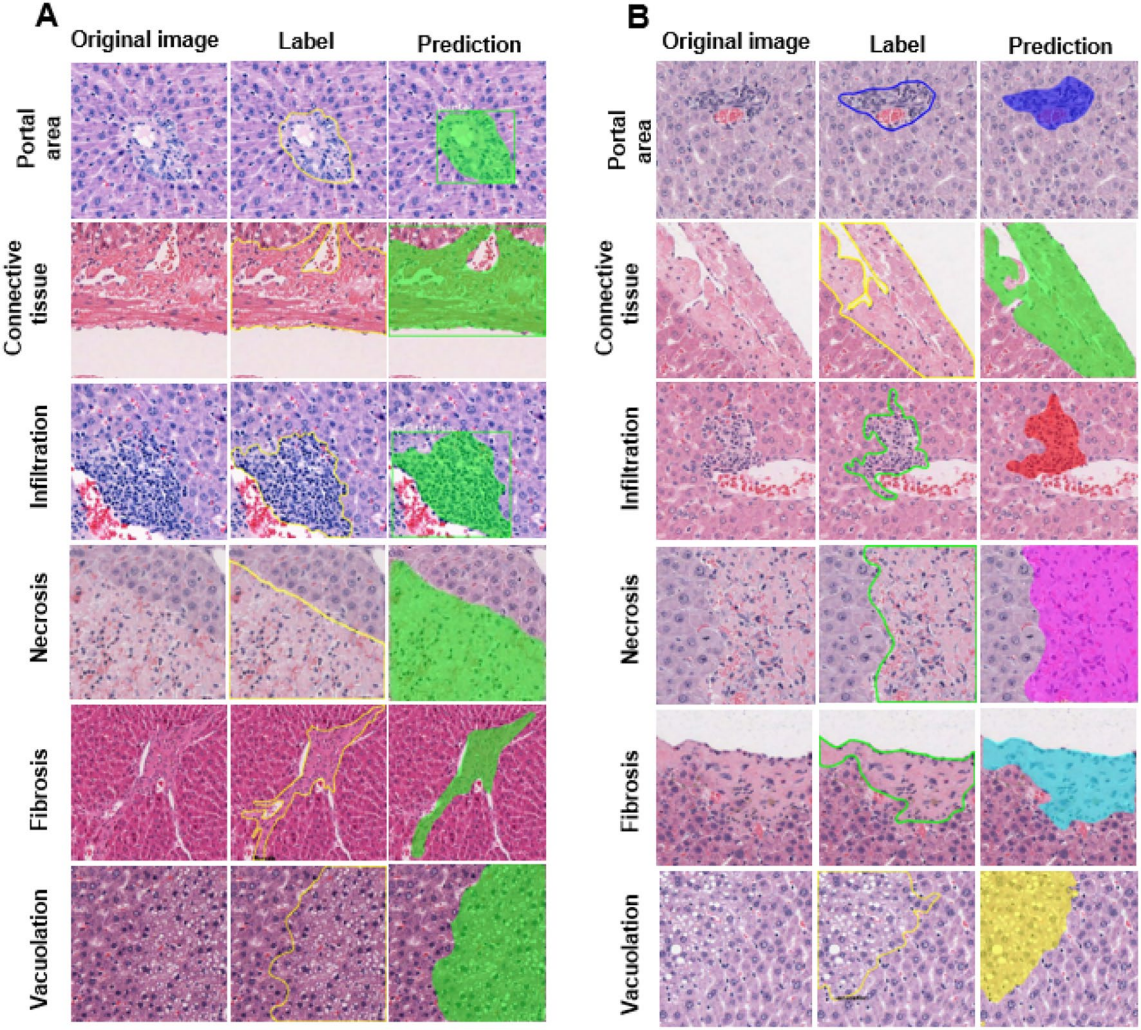
Comparison of original, labeled, and algorithm-predicted images in the single-finding models (**A**) and multiple-finding model (**B**). Left: original image tiles. Middle: labeled images for training of portal area, connective tissue, infiltration, necrosis, fibrosis, and vacuolation. Right: predicted area for each algorithm-determined lesion or normal feature.

The procedures used to establish the SFMs and MFM for liver injury are shown schematically in Fig. 1A,B, respectively. For each SFM, the algorithm was trained for one lesion or normal feature. Therefore, a total of six SFMs were trained. In contrast, the MFM was trained to include all six findings in a single model. For the MFM and each SFM, the total loss decreased steadily during the training, indicating that training was accomplished in all cases (Fig. 2A,B). Figure 3A,B show representative comparisons of original tile images, labeled images and the identifications made by the trained single-finding models and multiple-finding model for portal area, connective tissue, infiltration, necrosis, fibrosis, and vacuolation. In all cases, the algorithm effectively distinguished among the trained lesions and normal features in the image tiles. The predicted and labeled hepatic findings showed good overlap (see Fig. 3A,B, middle and right panels). To validate the established model after training, we tested the model accuracy by generating mean average precision (mAP) values. For the SFMs, the mAP values were 97.26%, 88.59%, 87.35%, 98.87%, 93.44%, and 88.32% for portal area, connective tissue, infiltration, necrosis, fibrotic lesion, and vacuolation, respectively. For the MFM, the overall mAP value was 92.46% and those for portal area, connective tissue, infiltration, necrosis, fibrosis, and vacuolation were 95.89%, 86.58%, 92.53%, 98.88%, 90.16% and 91.97%, respectively.

### Assessing SFM performance with respect to slide images

**Figure 4:**
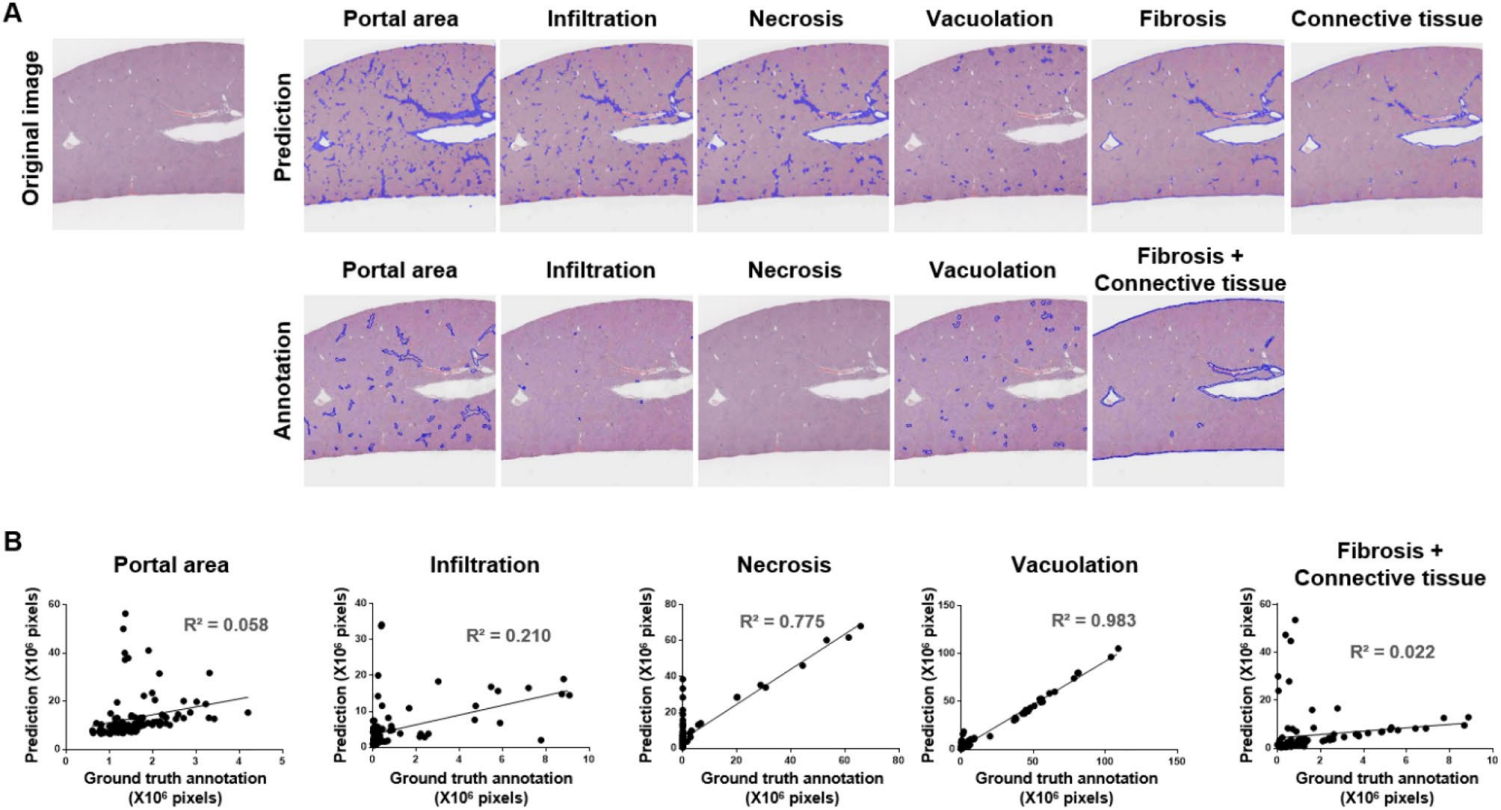
Comparison of original, annotated, and algorithm-predicted images in the single-finding models. (**A**) Portal area, connective tissue, infiltration, necrosis, fibrosis, and vacuolation in each algorithm prediction and ground-truth annotation. (**B**) Linear regression was used to compare the number of pixels annotated by an accredited toxicologic pathologist against those predicted by the established single-finding models for portal area, infiltration, necrosis, vacuolation, and connective tissue (including fibrosis).

To perform real-world testing of the trained SFMs, we assessed their ability to predict hepatic lesions and normal features in a total of 100 slide images, each of which corresponded to one third of a whole-slide image. Each SFM was used to predict the corresponding findings (Fig. 4A, blue color). In the slide-image setting, the similar appearance of connective tissue and fibrosis made it difficult for the pathologist to discriminate between these two findings; therefore, connective tissue and fibrosis were combined for ground-truth annotation. The ground-truth annotation was performed at 20 × magnification, and regions of portal area, infiltration, necrosis, vacuolation, and connective tissue (including fibrosis) were determined and recorded as the number of pixels involved (Fig. 4A, lower panel, blue color). As shown in Fig. 4A, all lesions and normal features except for vacuolation showed considerable differences between the SFM predicted and ground-truth annotated areas. Correlation analysis revealed that the results of the vacuolation SFM were highly correlated with those of the ground-truth annotation, whereas the other SFM predictions did not show good correlation with the ground-truth annotations (Fig. 4B).

Several deficiencies were found in the SFM prediction results, as shown in supplementary material S3. Portal area was generally overestimated, with the overestimation being particularly marked in NDMA- or APAP-treated animals. Necrosis was widely confused with connective tissue, and infiltration was greatly overestimated in APAP-treated animals. Connective tissue was confused with fibrosis and vice versa, and the combined area of these features was greatly overestimated in NDMA-treated animals. Vacuolation, however, was effectively predicted overall relative to the annotation (Supplementary material S3 and Fig. 4B).

Next, we analyzed the correlation between each SFM-based prediction and the annotation area in each treatment group (Supplementary material S4). Interestingly, for the SFM predicting connective tissue (including fibrosis), the results from NDMA-treated animals showed a high correlation (R^2^ = 0.914) with the annotated results; by contrast, the combined group (all treatment groups plus controls) showed a low correlation (R^2^ = 0.022) (Supplementary material S4A and Fig. 4B). For the SFM prediction of necrosis, the results from APAP-treated animals showed a higher correlation (R^2^ = 0.987) with the annotated results than did the combined group. For the SFM prediction of infiltration, no strong correlation was observed (Supplementary material S4B and Fig. 4B). For the SFM of vacuolation, the correlations between the prediction and annotation results were higher for the corn oil-treated group and combined group than for the other groups (Supplementary material S4C and Fig. 4B).

### Assessing MFM performance with respect to slide images

**Figure 5:**
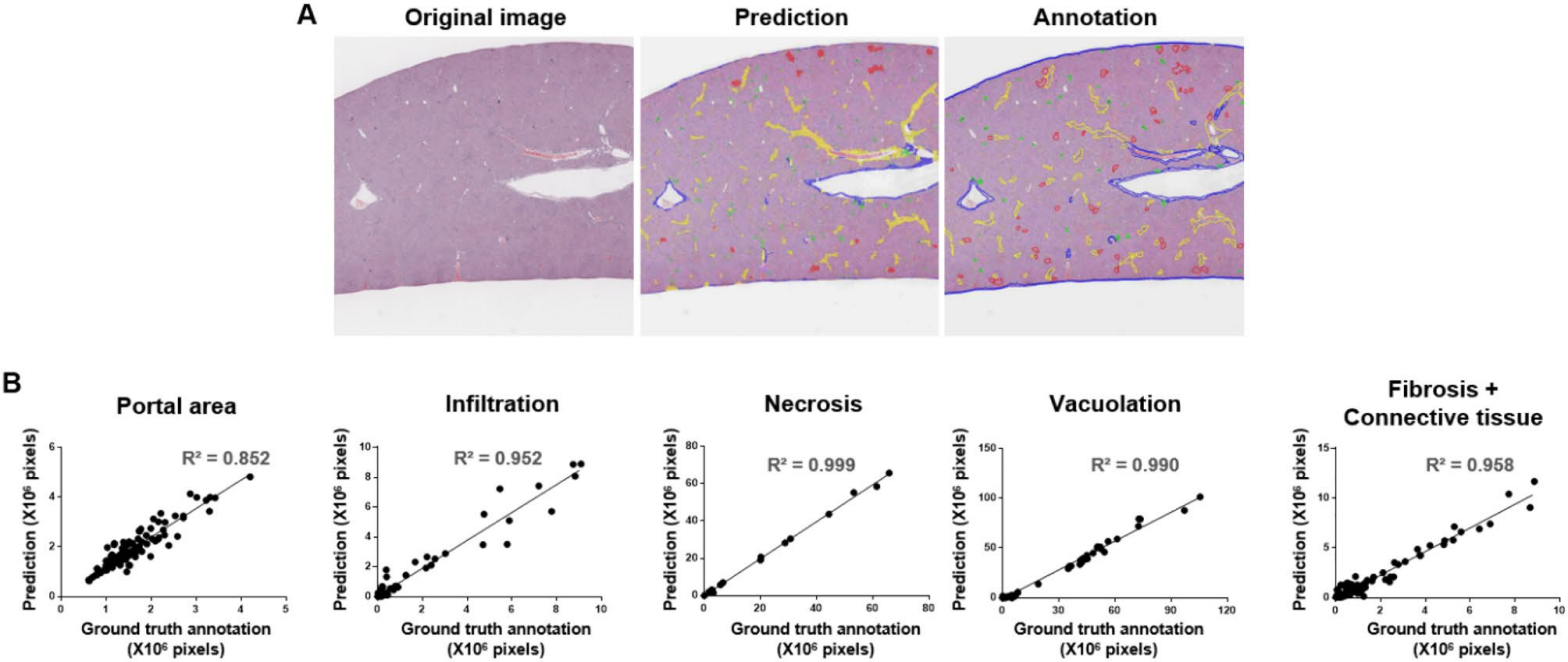
Comparison of original, annotated, and algorithm-predicted images in the multiple-finding model. (**A**) Portal area, connective tissue, infiltration, necrosis, fibrosis, and vacuolation in the algorithm prediction and ground-truth annotation. Representative slide images include portal area, connective tissue (including fibrosis), infiltration, necrosis, and vacuolation. Colors are as follows: portal area (yellow), connective tissue (blue), infiltration (green), and vacuolation (red). (**B**) Linear regression was used to compare the number of pixels annotated by an accredited toxicologic pathologist with those predicted by the established multiple-finding model for portal area, infiltration, necrosis, vacuolation, and connective tissue (including fibrosis).

Next, the MFM was used to predict liver lesions and normal features in the same slide images. In the predicted and annotated images, different colors were used to distinguish the type of feature: portal area (yellow), infiltration (green), necrosis (white), connective tissue plus fibrosis (blue), and vacuolation (red). The results obtained using the MFM showed patterns comparable to those of the ground-truth annotation (see representative prediction-merge image in Fig. 5A). However, due to the high complexity of multiple findings, several lesions were observed together in the same areas. These overlapping areas were classified into relevant findings by prioritizing the findings in the order of necrosis, connective tissue (including fibrosis), infiltration, portal area, and vacuolation. Correlation analysis revealed that the predicted areas of portal area, infiltration, necrosis, vacuolation, and connective tissue (including fibrosis) were highly correlated with the annotated dimensions, with all R^2^ values > 0.85 (Fig. 5B).

### Comparison of SFMs with MFM

**Figure 6:**
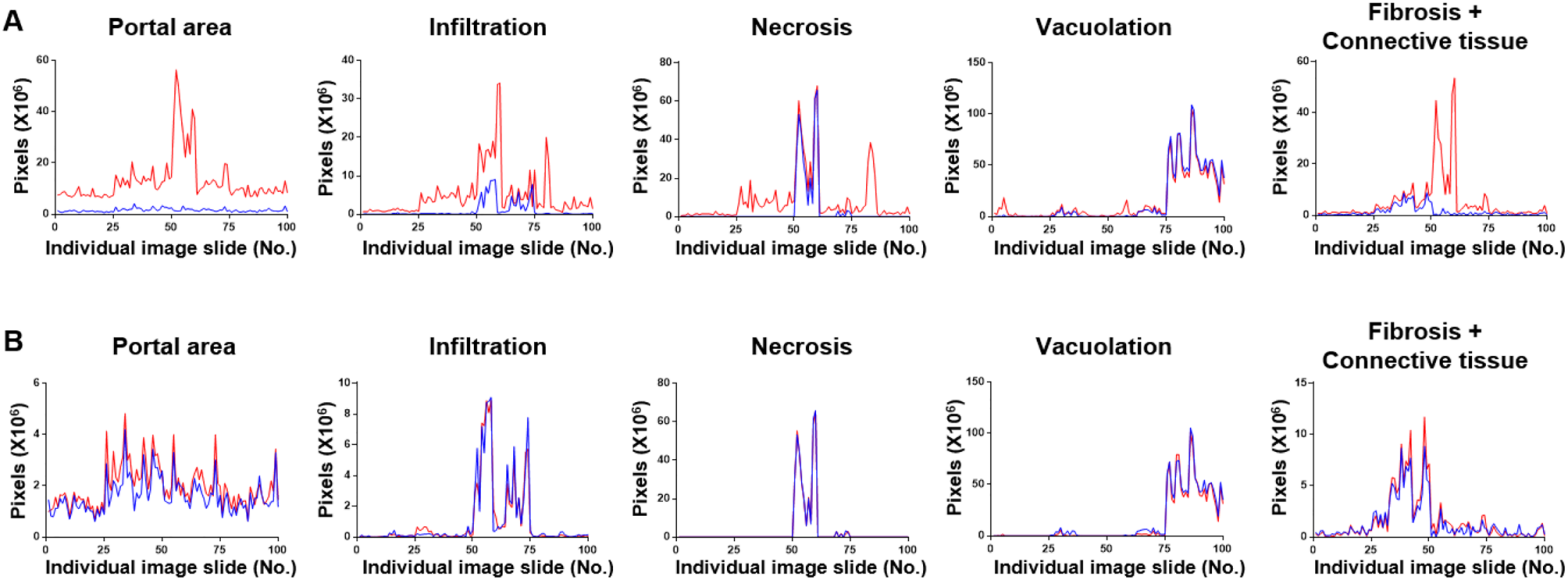
Comparison of the annotated and algorithm-predicted areas in the single- and multiple-finding models. (**A**) Blue lines indicate the ground truth-annotated area while red lines represent the algorithm-predicted area for portal area, infiltration, necrosis, vacuolation, and connective tissue (including fibrosis) in each single-finding model. (**B**) Blue lines indicate the ground truth-annotated area while red lines indicate the algorithm-predicted area for portal area, infiltration, necrosis, vacuolation, and connective tissue (including fibrosis) in the multiple-finding model.

We compared the SFM and MFM predictions at the individual slide-image level. For the SFMs, only the vacuolation SFM showed a pattern comparable with that of the ground-truth annotation, whereas the portal area, infiltration, and necrosis, and connective tissue (including fibrosis) SFMs generally overestimated or misestimated the areas of the features for which they were trained compared to the annotated areas (Fig. 6A). In contrast, the MFM exhibited patterns comparable to those of the annotated areas for all lesions and normal features (Fig. 6B). Thus, the results of the correlation analysis and individual slide-image analysis suggest that the MFM could be useful for predicting complicated lesions and normal features in a real-world setting.

## Discussion

In the present study, we applied Mask R-CNN-based deep learning to assess several types of liver lesions and normal features in a non-clinical study setting. The models were established through training and validation for single or multiple hepatic findings and the performance was assessed by comparison with pathologist-annotated slide images. Additionally, we compared the set of SFMs with the MFM. Each SFM yielded a mAP value above 85%, while the total mAP for the MFM was 92.46%, suggesting that the models had been successfully established. Compared to the SFMs, the MFM showed a higher correlation with the ground-truth annotation results assigned by an accredited toxicologic pathologist. Our results suggest that this new Mask R-CNN-based algorithm could be a useful tool that applies deep-learning to digital pathology, and that the MFM is more useful than individual SFMs in predicting different and complicated liver lesions and normal features.

All of the slide image results of the SFMs and MFM are shown in Supplementary material S1. The MFM, which predicted all trained lesions and normal features, showed a good correlation with the ground truth-annotated area. Among the SFMs, however, only the SFM for vacuolation yielded good slide image predictions. During training, connective tissue was mainly trained with slides from control animals and fibrosis was mainly trained with the slides from NDMA-treated animals; therefore, the established models showed high accuracy values for these treatment groups. However, the model failed to accurately discriminate between these findings, and exhibited confusion between them in the other groups (Figs. 4 and 6A). We analyzed the correlation between each SFM-based prediction and the corresponding annotation area in each treatment group, as shown in Supplementary material S4. These results suggest that each SFM could be useful for predicting toxicity under certain circumstances, but would not be appropriate in general or for more complex findings. Given that histopathological lesions in acute and chronic liver injuries are complicated, with multiple lesions often observed together^20–22^, our results indicate that the MFM could be useful for predicting real-world histopathological liver lesions.

The histological spectrum of DILI is broad^23^ and image analysis of various hepatic lesions related to liver injury and liver toxicity is considered to be critical for the diagnosis and severity assessment of this disease^24^. Recently, deep learning based on deep neural networks has been applied to the assessment and diagnosis of various lesions in non-clinical and clinical studies^25,26^. The use of deep-learning algorithms in non-clinical studies can allow rapid, quantitative, and consistent histopathological assessment in novel drug development processes and toxicological studies with implications for clinical settings. Therefore, a classification network capable of detecting and discriminating particular lesions may be favored over object detection and/or segmentation, which require high calculation loads and labor-intensive annotation procedures. Deep learning-based diagnosis or quantification of liver lesions has been attempted in several studies^27–29^. Puri tested the ability of automated machine-learning models to classify DILI injury patterns from whole-slide images, and obtained an average precision of 98.6%^30^. Xu et al*.* employed deep learning for DILI prediction in response to 198 drugs and observed good accuracy, sensitivity, and specificity^2^. In addition to these studies, there are several reports on the performance of the model in the dataset environment; however, AI-based pathological analysis has not yet been sufficiently studied using real-world slide images^31^. In the present study, we confirmed the possibility of applying AI-assisted image analysis in a drug-induced liver injury rat model. The results suggested that the proposed trial has potential for use in clinical applications. However, the proposed approach was not validated in human samples, untrained complex lesions, severity scoring, or various real-world settings. Moreover, in the present study we compared the number of pixels, not the actual concordance of pixel location between ground truth annotation and model prediction. Therefore, further studies will be needed before the method can be applied to human diagnosis.

Here, we used a Mask R-CNN algorithm to assess hepatic lesions induced by NDMA, APAP or corn oil in rats. The study results suggest that the Mask R-CNN-based MFM could be a useful tool for detecting and predicting multiple and complicated toxic liver lesions in basic non-clinical study settings.

## Materials and methods

### Animal study

Sprague–Dawley (SD) rats (7 weeks of age, males and females) were obtained from Orient Bio, Inc. (Republic of Korea). The rats were maintained at a temperature of 23 ± 3 °C and a relative humidity of 30–70% under a 12 h light/dark cycle, and allowed to acclimate for 7 days prior to the first administration of drug or vehicle. All experimental procedures involving animals were approved by the Assessment and Accreditation of Laboratory Animal Care International (AAALAC) and Institutional Animal Care and Use Committee (IACUC). All animal experiments were conducted in accordance with the ARRIVE guidelines and all methods were carried out in accordance with the principles and procedures outlined in the National Institutes of Health (NIH) Guide for the Care and Use of Laboratory Animals. The rats were randomly assigned to four groups: control (distilled water), N-nitrosodimethylamine (NDMA)-treated, acetaminophen (APAP)-treated, and corn oil-treated. The animal study design and liver tissue slide preparation procedure are detailed in Table 1. Necropsy was performed at 24 h after the last dosing, and liver tissues were collected and fixed in 10% neutral buffered formalin. Each collected liver was divided into pieces, which were paraffin-embedded and used to prepare slides.

### Slide image preparation

A total of 100 liver samples representing 25 sections per group were H&E-stained and digitized into slide images using an Aperio XT slide scanner (Leica Biosystems, Wetzlar, Germany). Slide images of liver sections were scanned using an Aperio ScanScope XT (Leica Microsystems, USA) with a 20 × objective and bright-field illumination. The scan resolution was 0.4993 μm per pixel and the images were saved as JPEG image-compression files. The data were prepared for detection of portal area, connective tissue, vacuolation, infiltration, necrosis, and fibrosis, as previously described^19^. Briefly, the slide images (20×-magnified) were cropped into 448 × 448 pixel tiles, and VGG Image annotator 2.0.1.0 (Visual Geometry Group, Oxford University, UK) was used to label all lesions and normal features. An accredited toxicologic pathologist assessed the annotated lesions and normal features, and verified findings were labeled and used to first train and then test the Mask R-CNN algorithm. To distribute the annotated image tiles into training, validation, and test datasets (approximately 7:2:1), we used the train_test split function of the scikit-learn package. To improve the training dataset, we used data augmentation: eight-fold augmentation was enabled using image-augmentation techniques such as reversal, rotation, and brightening. For SFM and MFM, total datasets of 46,284 and 48,772 image tiles, respectively, were used to train, validate, and test the algorithm on liver injury lesions and normal features. The numbers of image tiles used for each training, validation, and testing set are presented in Supplementary material S5.

### Mask R-CNN algorithm

All algorithm-training procedures, including the distribution of data, were undertaken as previously described^19^. Briefly, an open-source framework for machine learning (Tensorflow 2.1.0 with a Keras 2.4.14 backend) powered using an NVIDIA RTX 3090 24G GPU was used for training, along with Matterport Mask R-CNN 2.1 (Sunnyvale, CA, USA). The Mask R-CNN algorithm (https://github.com/matterport/Mask_RCNN) consisted of two stages: first, a region proposal network (RPN) proposed candidate object-bounding boxes; and second, RoIAlign extracted features that would be used to predict pixel-accurate masks. In RoIAlign, bi-linear interpolation is applied to determine the exact values of input features at four regularly sampled locations for each RoI bin, and the results are aggregated using max pooling.

### Model training, validation, and testing

In the SFMs, four lesions and two normal features were trained on separate Mask R-CNNs to create a model for each lesion and normal feature. The MFM was created as a single model by learning all four lesions and two normal features in one Mask R-CNN. The hyperparameters applied for training are presented in Table 2. The default settings of the Matterport package were used for all configurations, except for five parameters that were customized to fit the hepatic injury dataset. IMAGE_PER_GPU was used to simultaneously analyze four images, and the training utilized four GPUs. The 448 × 448 image size was determined from IMAGE_MAX_DIM and IMAGE_MIN_DIM based on the size of the slide image. The instance classification accuracy threshold, which was called DETECTION_MIN_CONFIDENCE, was set at 0.5. For determination of the Mask R-CNN loss (total loss), we summed the losses (including the smooth L1 loss for the bounding box, the sparse softmax cross-entropy loss for the label, and the binary cross-entropy loss for the mask). To verify the model performance, the mAP was calculated based on the intersection of the precision, union (IoU), and recall values. The IoU value, which was calculated according to a previous report^19^, reflects the ratio of the area overlaid by the union of the predictions to that overlaid by the ground truth. The mAP value, which reflects model accuracy, was generated. We used the transformed mAP, which goes to 0 when an image is identified as containing a misprediction. This transformation was used to analyze the error cases in more detail, investigate the basis for correct and incorrect predictions, and evaluate the model performance more strictly.

**Table 2:** Hyperparameters used in Mask R-CNN training of single- and multiple-finding models.

Hyperparameter	Value
Backbone	resnet152
Dist_backend	"nccl"
Batch_size	16
LR	0.005
Epochs	200
Workers	16
LR_steps	16, 22
Confidence	0.5
mIoU confidence	0.667

### Slide image confirmation

One-hundred slide images were used as the confirmation set. Each slide image, which was 10,752 × 10,752 pixels and had not been used during the training, was cropped by Aperio Image Scope version 12.4.0 (Leica Biosystems, USA) from whole-slide images that were scanned by an Aperio ScanScope XT (Leica Biosystems, USA) using a 20 × objective and bright-field illumination. Before being submitted for confirmation, each hepatic lesion, normal feature, and tissue type was annotated by an accredited toxicologic pathologist; this was used as the ground truth that would later be compared with the algorithm-based prediction. The ground-truth annotations for all lesions and normal features were applied using VGG Image annotator 2.0.1.0 (Visual Geometry Group, Oxford University, UK), as described for the annotation of findings used in model training. The annotated areas were calculated based on pixel counting. Each cropped slide image was divided into tile images of 448 × 448 pixels. The trained algorithm was used to predict each hepatic lesion and normal feature, and the prediction mask-bearing cropped images were merged into an overall slide image. Linear regression was used to compare the calculated prediction mask areas with the ground truth annotated areas. The histopathological annotations used to label the images are shown in Supplementary material S6.

### Supplementary Information

Supplementary Information.

## Data Availability

The image datasets generated during the current study were deposited into public repository (10.5281/zenodo.8423742).
