# Supplementary material for: Application of multiple-finding segmentation utilizing Mask R-CNN-based deep learning in a rat model of drug-induced liver injury
Source: Sci Rep. 2023 Oct 16;13:17555. doi: 10.1038/s41598-023-44897-8 (PMC10579263; doi:10.1038/s41598-023-44897-8)
Supplement: Supplementary file 1 — Supplementary Information. [file 41598_2023_44897_MOESM1_ESM.pdf]

Application of multiple-finding segmentation utilizing Mask R-CNN-based deep learning in a rat model of drug-induced liver injury

Eun Bok Baek[1#], Jaeku Lee[2#], Ji-Hee Hwang[3], Heejin Park[3], Byoung-Seok Lee[3], Yong-Bum Kim[4], Sang-Yeop Jun[2], Jun Her[2], Hwa-Young Son[1*], Jae-Woo Cho[3*]

Supplementary material 1. Slide image results of annotation, SFM and MFM

Group	No. of slide images	Ground truth annotation (pixels)					SFM prediction (pixels)					MFM prediction (pixels)				
		Portal area	Infiltration	Necrosis	Vacuolation	Fibrosis + Connective tissue	Portal area	Infiltration	Necrosis	Vacuolation	Fibrosis + Connective tissue	Portal area	Infiltration	Necrosis	Vacuolation	Fibrosis + Connective tissue
	1	1,454,113	23,248	0	47,073	457,774	7,508,969	690,668	560,290	3,349,912	851,644	1,001,939	16,315	0	7,777	297,026
	2	820,060	136,917	0	11,925	170,615	7,726,366	670,994	648,974	2,083,764	744,132	1,057,498	54,341	0	0	146,140
	3	792,928	21,793	0	162,411	618,872	8,672,687	761,100	795,829	9,112,742	1,099,962	1,132,525	22,516	0	482,456	302,435
	4	1,125,707	22,766	0	45,029	628,012	8,873,306	1,324,645	1,607,225	7,142,908	1,764,731	1,560,252	13,888	0	0	540,364
	5	1,130,711	98,917	0	60,998	159,425	7,380,923	848,275	652,253	18,733,241	795,798	1,170,461	73,911	0	1,780,207	73,785
	6	1,505,730	0	0	64,362	143,546	7,172,476	935,536	540,603	7,565,559	667,303	1,655,594	16,622	0	184,299	61,173
	7	1,312,810	0	0	69,959	139,563	6,766,369	1,017,531	1,335,799	1,506,865	1,334,142	1,621,656	2,389	0	28,684	16,450
	8	1,601,469	0	0	45,647	326,065	7,302,416	973,487	1,550,325	1,475,850	1,319,600	1,727,148	0	0	35,429	127,360
	9	1,203,197	0	0	39,921	238,941	8,802,028	798,227	1,051,089	814,251	1,162,008	1,452,553	18,440	0	0	89,648
	10	695,221	19,778	0	175,950	489,573	10,888,915	693,308	1,455,428	3,243,012	914,730	787,241	16,985	0	40,237	365,089
	11	994,441	27,287	0	0	467,612	7,061,441	809,397	1,443,590	590,200	1,324,449	1,258,132	31,749	41,070	0	243,283
Control	12	1,624,283	72,528	0	0	279,037	7,784,586	1,006,377	1,406,641	258,616	1,274,415	1,758,102	50,870	0	0	173,371
	13	1,210,365	12,337	0	18,965	508,882	7,263,651	1,080,611	1,407,280	626,746	1,384,423	1,355,777	21,140	0	0	410,221
	14	1,100,735	278,043	0	0	439,602	7,630,324	1,399,478	1,326,585	463,346	1,170,801	1,600,181	205,179	0	0	482,378
	15	1,293,834	181,550	0	0	217,753	6,868,478	1,229,005	1,004,616	76,999	955,858	1,456,027	126,936	0	18,368	194,085
	16	1,430,509	444,296	0	14,023	1,039,891	10,176,980	1,647,926	2,854,568	62,903	2,295,955	1,493,782	198,500	0	8,754	1,186,219
	17	966,483	66,385	0	0	710,666	6,886,675	985,105	1,312,129	450,954	1,277,449	1,079,674	60,275	0	0	679,198
	18	1,200,637	162,921	0	0	399,806	7,196,368	1,279,673	1,022,383	149,670	1,250,272	1,565,239	62,994	0	0	318,739
	19	1,011,516	78,052	0	72,310	621,522	6,983,873	917,937	1,595,900	524,737	1,124,785	1,074,486	75,196	0	12,428	559,881
	20	1,011,168	79,902	0	0	255,400	6,421,843	951,816	544,230	15,449	648,201	1,218,636	78,891	0	0	183,886
	21	603,095	42,099	0	0	1,123,109	7,817,552	577,323	1,146,844	1,597,583	1,347,494	670,921	38,133	0	0	1,050,357
	22	1,094,121	89,089	0	0	588,744	7,538,406	964,917	877,318	41,856	1,289,137	1,213,669	75,998	7,058	0	557,565
	23	798,594	112,288	0	0	178,644	6,589,087	815,588	564,884	103,876	546,412	880,605	107,196	0	0	90,740
	24	1,189,576	98,947	0	47,474	755,639	7,085,979	1,127,447	836,989	278,896	1,352,970	1,361,108	97,765	0	0	704,722
	25	879,909	59,994	0	0	662,970	7,431,753	787,493	966,877	454,129	1,028,625	971,678	61,883	0	0	589,813
	26	2,865,098	230,335	0	1,053,398	2,402,515	15,347,280	5,645,363	6,707,230	3,931,405	4,364,726	4,136,983	667,334	37,913	647,145	1,604,949
	27	1,178,990	149,277	0	696,866	2,586,775	13,048,188	3,342,689	15,775,104	1,251,671	3,861,142	2,146,967	435,343	0	187,175	2,116,538
	28	1,571,423	170,156	0	4,245,865	1,218,904	10,490,662	2,906,638	6,201,633	6,325,580	2,441,666	1,817,066	479,747	0	1,357,717	919,388
	29	2,212,561	198,539	0	3,553,652	2,214,635	14,357,896	4,540,047	7,766,684	5,530,503	3,436,329	3,353,239	680,387	11,116	1,708,514	1,837,461
	30	2,019,379	208,157	0	8,073,045	926,549	11,342,516	4,275,400	4,423,629	12,102,396	2,556,893	2,331,321	654,318	17,649	5,676,094	877,646
	31	1,586,408	223,847	0	970,905	2,442,009	13,302,826	3,182,463	18,988,516	1,985,733	3,512,960	2,146,066	347,883	40,846	217,748	2,123,090
	32	1,990,250	188,783	0	2,332,752	1,114,309	10,227,700	3,322,503	2,380,706	3,792,428	2,345,258	2,749,551	406,885	0	910,239	525,320
	33	2,047,818	63,294	0	5,062,754	3,779,118	20,511,119	7,432,282	11,809,986	3,526,569	5,111,822	3,125,556	88,211	0	40,292	4,257,490
	34	4,197,618	168,423	0	1,078,463	5,242,882	15,260,380	5,753,339	7,880,700	2,651,841	7,898,277	4,821,992	214,137	11,568	100,153	5,811,725
	35	2,306,339	238,608	0	5,117,875	4,862,006	13,220,293	3,969,280	5,634,384	6,513,853	7,041,276	2,994,611	149,927	0	258,856	5,750,869
	36	2,530,872	36,350	0	4,686,877	3,745,222	12,621,962	4,979,479	4,911,701	8,789,410	5,727,248	3,252,776	50,081	0	432,785	4,288,003
	37	2,168,853	153,021	0	465,770	3,645,428	11,646,926	4,264,993	5,834,215	2,094,282	5,997,787	2,373,297	78,596	0	18,145	4,896,162
NDMA	38	1,023,792	247,908	0	308,953	8,677,577	14,902,127	4,517,804	9,579,923	2,818,184	9,649,699	1,981,098	149,894	0	0	9,083,103
	39	1,730,079	68,145	0	192,581	4,822,759	12,777,099	5,664,016	6,907,903	5,185,513	6,810,343	2,645,048	84,290	0	235,694	5,360,739
	40	1,507,033	26,095	0	0	6,890,439	14,092,121	5,401,032	7,538,792	4,139,647	8,356,178	2,212,453	30,314	0	0	7,429,826
	41	1,707,775	4,294	0	159,180	6,408,406	13,044,580	4,243,995	8,743,578	2,373,748	8,346,929	2,328,214	21,731	2,262	0	6,926,119
	42	3,223,925	7,187	0	11,008	7,722,349	18,832,878	7,413,926	13,806,989	657,729	12,726,460	3,884,644	65,661	0	0	10,434,932
	43	2,158,548	204,041	0	0	1,403,953	10,069,939	3,357,207	3,115,950	555,553	3,420,783	3,011,124	81,856	0	0	1,786,190
	44	1,424,313	49,600	0	0	2,108,922	9,518,140	1,974,162	2,377,546	420,823	3,087,632	1,872,986	57,755	0	0	2,528,308
	45	1,889,518	57,931	0	0	3,099,631	10,927,674	3,047,212	4,015,077	94,773	4,798,794	2,468,904	42,360	0	0	3,639,257
	46	3,421,561	145,195	0	0	2,617,581	12,663,228	4,938,062	5,916,214	12,103	5,861,914	3,979,008	125,294	0	0	3,562,395
	47	2,713,501	15,676	0	21,245	4,210,600	12,982,792	4,324,032	5,771,690	1,409,725	6,351,728	3,172,518	243,525	0	0	5,269,961
	48	2,713,435	713,911	0	0	8,865,244	20,072,428	8,230,971	13,035,569	1,122,258	13,026,800	3,250,272	460,667	0	0	11,700,288
	49	2,269,130	178,866	0	0	5,593,253	13,755,900	3,831,843	7,023,064	273,698	7,634,431	2,686,121	67,299	0	0	6,626,549
	50	2,585,492	439,172	0	0	5,292,736	14,484,575	5,117,014	8,503,039	24,857	8,734,527	2,429,208	114,895	0	0	7,150,312

(Continued)

Group	No. of slide images	Ground truth annotation (pixels)					SFM prediction (pixels)					MFM prediction (pixels)				
		Portal area	Infiltration	Necrosis	Vacuolation	Fibrosis + Connective tissue	Portal area	Infiltration	Necrosis	Vacuolation	Fibrosis + Connective tissue	Portal area	Infiltration	Necrosis	Vacuolation	Fibrosis + Connective tissue
APAP	51	1,429,435	3,027,117	30,670,658	0	55,358	37,939,123	18,365,043	33,973,594	952,238	24,070,170	1,544,575	2,896,282	30,466,909	22,812	793,797
	52	1,365,247	5,786,593	53,240,493	198,894	618,002	56,366,539	15,690,147	60,321,266	956,679	44,897,300	1,850,433	3,521,014	55,295,162	0	1,334,447
	53	1,318,463	1,672,429	44,366,674	0	35,223	50,008,136	10,901,709	46,103,394	1,576,780	30,108,190	1,484,738	2,325,518	43,707,057	0	199,363
	54	1,351,822	7,195,726	28,757,825	161,088	547,009	40,023,492	16,551,156	35,349,845	3,589,620	28,005,130	1,954,762	7,430,431	28,276,918	68,355	1,475,608
	55	3,308,878	5,462,470	20,030,659	38,930	2,792,055	31,749,372	16,842,266	28,367,186	2,259,381	16,746,480	4,003,916	7,230,355	19,106,759	18,736	3,357,051
	56	1,792,059	8,737,103	5,737,809	0	708,906	22,200,583	14,826,768	13,166,491	3,459,370	8,147,330	1,823,207	8,869,861	5,807,133	0	1,352,431
	57	2,151,530	8,820,531	20,168,876	291,656	1,612,458	31,488,039	19,016,682	28,619,238	9,422,356	16,060,830	2,310,469	8,077,542	20,703,151	116,240	1,692,544
	58	1,984,473	9,085,540	6,697,092	1,019,105	485,641	23,418,225	14,511,676	14,045,989	16,331,020	8,427,494	1,621,529	8,899,926	7,011,893	388,391	1,467,659
	59	1,900,558	385,310	61,400,129	0	383,091	41,091,341	33,784,827	61,736,284	3,583,119	47,439,290	2,121,354	1,806,293	58,491,909	70,546	1,299,351
	60	1,355,967	403,543	65,803,686	0	826,190	37,218,865	34,159,766	68,082,721	1,370,522	53,609,120	1,576,667	1,340,721	65,690,517	18,771	1,068,631
	61	1,436,923	686,726	0	1,084,396	229,476	7,689,872	1,857,415	2,032,601	3,375,654	1,409,467	1,722,655	562,435	16,617	150,271	128,684
	62	1,112,492	560,752	0	0	834,752	9,281,293	1,603,302	1,752,218	571,738	1,511,394	1,355,365	554,818	0	0	758,528
	63	1,793,143	793,270	0	4,595,571	456,862	10,181,205	4,644,767	2,253,750	5,370,421	2,425,091	2,206,379	710,442	0	2,024,651	922,992
	64	2,294,811	916,512	0	5,003,680	955,689	10,824,273	4,864,214	3,272,454	5,162,578	3,405,279	2,484,624	648,462	0	1,993,237	1,311,891
	65	1,771,367	4,710,643	0	5,909,495	225,507	13,187,607	7,640,699	5,257,031	4,947,717	4,518,392	2,721,453	3,488,055	18,360	1,575,573	1,305,069
	66	2,090,764	2,398,961	0	6,396,030	467,862	11,490,693	2,842,273	3,215,203	10,786,421	2,566,768	2,236,973	2,117,844	0	1,957,306	996,692
	67	1,739,226	2,163,432	0	6,545,653	1,326,523	12,243,079	2,936,288	3,758,433	10,451,281	3,655,890	2,627,936	1,929,785	0	1,930,964	1,511,014
	68	1,503,513	5,878,431	0	5,472,642	95,567	13,525,286	6,773,771	3,864,844	9,369,898	3,264,258	2,112,387	5,104,442	0	944,406	864,960
	69	1,128,556	1,265,748	2,866,888	3,247,757	1,059,494	12,833,311	3,861,489	6,321,379	4,656,743	3,919,214	1,692,780	1,445,303	2,989,053	1,380,101	740,194
	70	1,293,349	2,566,509	0	7,912,206	501,315	10,611,837	3,783,302	2,159,036	10,783,360	2,002,223	1,608,952	2,538,487	0	4,881,671	572,852
	71	1,728,131	851,109	1,625,251	2,153,524	637,002	10,505,401	5,957,456	3,910,818	3,390,826	3,694,115	2,034,203	724,856	1,893,009	775,833	424,892
	72	1,644,724	2,209,485	0	5,131,441	555,966	11,073,437	3,775,998	1,531,194	8,279,115	1,926,307	1,600,581	2,670,318	0	2,815,531	594,696
	73	3,012,892	4,753,681	3,277,334	1,486,003	1,686,067	19,763,107	11,548,076	9,927,309	2,834,512	8,592,109	3,996,015	5,526,530	1,470,350	146,089	2,087,844
	74	1,173,458	7,777,249	2,587,514	1,686,991	882,328	19,512,656	2,012,519	8,508,386	3,029,169	7,052,707	2,092,149	5,720,274	3,010,371	495,932	2,162,081
	75	1,631,201	425,699	0	1,408,089	809,050	9,586,455	11,586,577	1,480,886	3,043,936	1,582,287	2,003,854	273,544	0	563,978	613,532
Corn-oil	76	759,665	76,733	0	61,184,350	165,366	7,370,512	1,497,278	2,853,650	60,251,260	526,928	861,375	91,770	0	59,180,692	111,571
	77	1,449,305	56,759	0	72,481,671	974,690	8,720,039	2,923,391	5,579,097	74,253,998	1,927,695	2,103,761	33,656	0	72,098,206	449,848
	78	1,735,334	23,096	0	42,228,749	1,321,203	9,781,023	2,202,797	2,511,156	38,250,815	2,686,439	1,858,102	10,150	0	35,873,866	1,070,728
	79	996,098	117,563	0	36,580,833	962,575	9,559,523	1,699,106	2,951,534	33,294,910	1,776,943	1,446,022	94,407	0	32,084,000	565,481
	80	806,968	239,070	0	72,726,534	638,525	10,316,487	20,023,806	4,817,795	79,945,006	823,096	1,105,029	98,905	0	79,339,427	294,906
	81	1,186,716	236,721	0	73,521,575	328,736	10,189,860	14,256,425	6,085,663	80,058,122	1,212,761	1,602,312	77,877	0	79,196,917	124,469
	82	848,283	39,960	0	48,291,085	186,427	6,772,801	1,688,051	28,402,495	45,500,462	984,433	967,085	43,742	0	45,107,139	134,606
	83	1,293,279	49,371	0	42,336,587	920,261	11,354,601	2,108,495	38,555,279	37,746,585	1,946,889	1,675,034	37,947	0	37,943,580	602,210
	84	1,502,625	38,671	0	44,948,381	138,423	6,941,114	1,220,531	33,386,708	43,423,798	1,043,368	1,262,505	32,221	0	42,819,229	161,592
	85	1,125,922	46,084	0	41,520,661	305,816	7,163,566	1,041,136	21,361,835	38,335,751	1,130,210	1,411,357	52,025	0	37,551,538	219,636
	86	1,269,756	31,211	0	105,444,484	147,363	9,235,357	891,345	1,872,283	105,450,973	506,868	1,415,792	0	0	101,584,765	114,000
	87	619,517	39,516	0	97,033,080	391,195	6,845,947	489,365	454,794	96,624,251	563,017	637,243	12,954	0	87,919,791	280,112
	88	1,728,198	327,092	0	56,301,911	239,600	9,971,617	3,282,080	1,581,493	58,701,463	1,104,318	1,961,989	157,368	0	56,838,386	98,622
	89	1,109,262	345,976	0	50,738,092	1,249,148	10,415,465	2,363,933	3,274,845	52,778,046	930,362	1,644,896	129,023	0	51,559,251	156,740
	90	1,519,646	140,570	0	42,773,498	252,529	8,438,370	1,295,347	1,168,812	41,225,237	1,179,780	1,262,969	68,488	0	39,503,321	419,465
	91	1,521,596	89,483	0	43,680,436	254,816	8,035,869	1,430,074	2,014,521	41,086,505	1,071,896	1,807,311	37,224	0	38,610,853	193,727
	92	2,390,439	22,586	0	54,399,262	0	10,112,625	4,588,103	1,781,532	49,840,601	1,516,804	2,061,073	6,726	8,678	46,088,284	81,662
	93	1,637,196	32,518	0	41,293,812	385,235	8,714,164	1,509,970	1,366,004	37,439,374	1,301,753	1,666,911	3,374	0	33,626,761	357,778
	94	1,308,053	51,961	0	50,505,595	111,610	7,991,055	1,749,247	1,884,786	52,649,942	746,363	1,267,946	40,415	0	50,680,103	67,686
	95	1,311,852	98,470	0	52,111,121	110,686	10,825,274	3,320,520	2,427,023	52,655,480	849,315	1,401,219	12,504	0	51,362,590	89,111
	96	1,180,382	172,963	0	45,201,253	347,117	8,932,301	2,332,538	995,931	40,091,605	1,041,634	1,576,298	107,255	0	39,184,595	173,819
	97	1,378,598	87,018	0	35,017,614	893,808	9,229,138	2,293,763	2,296,285	30,381,858	2,218,249	2,199,087	76,440	0	29,303,445	710,940
	98	1,392,449	153,447	0	19,023,301	982,960	8,087,024	1,915,263	1,759,542	13,834,593	1,742,445	1,777,087	60,242	18,796	13,881,400	827,074
	99	3,295,116	139,233	0	52,491,615	1,223,897	13,088,990	4,412,291	5,088,095	49,450,323	3,924,456	3,437,782	12,502	0	49,279,172	1,355,797
	100	1,187,386	164,298	0	35,574,389	283,879	8,514,588	1,507,991	1,466,729	31,946,388	886,435	1,455,086	70,652	0	31,691,934	140,993

Supplementary material 2.

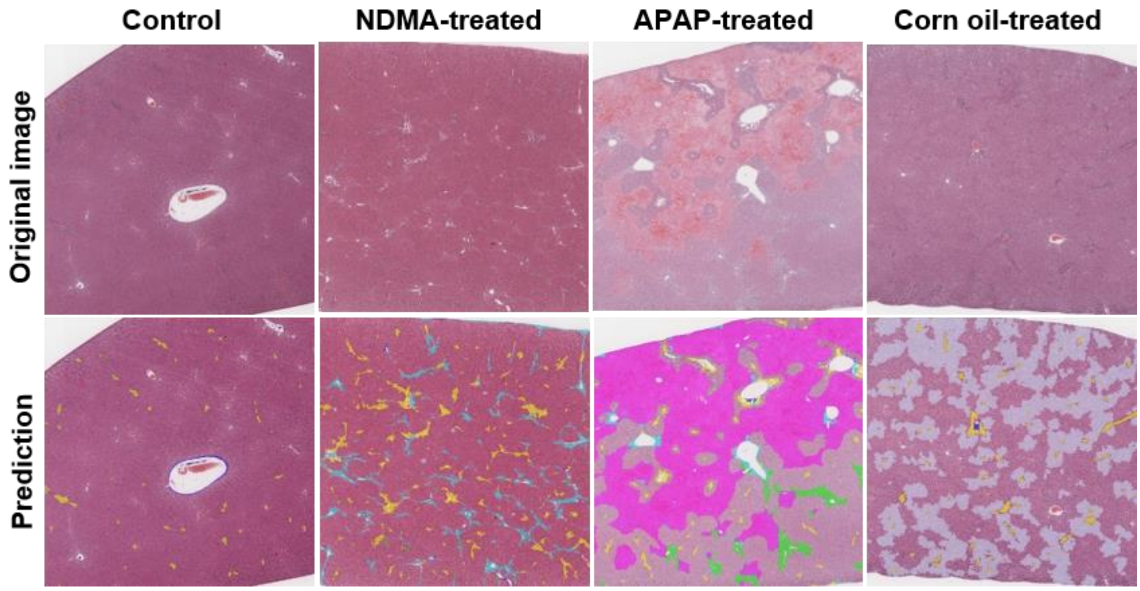

Supplementary material 2. Representative original and algorithm-predicted images of the multiple-finding model in each treatment group. Upper and lower panels present original slide images and algorithm-predicted images, respectively, of the multiple-finding model for animals of the control, NDMA-, APAP-, and corn oil-treated groups.

Supplementary material 3. Confusion summary in SFM

Lesion or normal feature	Confusion points
Portal area	Overestimated; highly overestimated in NDMA- or APAP-treated animals
Necrosis	Highly confusion with connective tissue
Infiltration	Highly overestimated in APAP-treated group
Connective tissue	Confusion with fibrosis
Fibrosis	Confusion with connective tissue
Connective tissue (including fibrosis)	Highly overestimated in NDMA-treated group

Supplementary material 4.

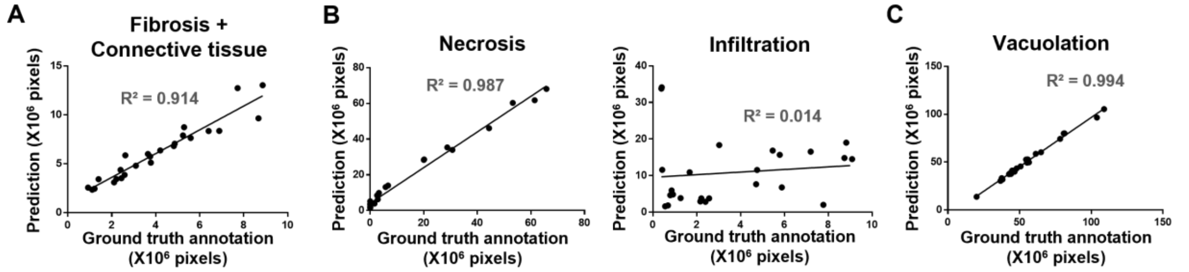

Supplementary material 4. Comparison of the annotated and algorithm-predicted areas in the single-finding models. (A) For slide images from NDMA-treated animals, connective tissue (including fibrosis) area was compared by linear regression between the annotation and single-finding model prediction. (B) For slide images from APAP-treated animals, necrosis and infiltration area were compared by linear regression between the annotation and single-finding model prediction. (C) For slide images from corn oil-treated animals, vacuolation area was compared by linear regression between the annotation and single-finding model prediction.

.

Supplementary material 5. Number of cropped tile images for the model training, validation, and testing

		Portal area	Connective tissue	Infiltration	Necrosis	Fibrosis	Vacuolation
**Single-finding models**	Training	943 (7,544)	1,050 (8400)	1,159 (9,272)	573 (4,584)	737 (5,896)	1,032 (8,256)
	Validation	254	288	335	147	214	272
	Testing	154	166	191	90	65	156
	Total	7,952	8,854	9,798	4,821	6,175	8,684
**Multiple finding model**	Training	1,239 (9,912)	1,050 (8,400)	1,159 (9,272)	573 (4,584)	737 (5,896)	1,032 (8,256)
	Validation	332	288	335	147	214	272
	Testing	196	166	191	90	65	156
	Total	10,440	8,854	9,798	4,821	6,175	8,684

Numbers in brackets indicate data augmentation using a randomized combination of image-augmenting techniques, including reversal, rotation, and brightening

Supplementary material 6. Criteria for histopathological annotations

Lesion or normal feature	Criteria
Necrosis	Liver areas with pale cytoplasm and pyknotic or karyolytic nuclei, also labeled with scattered blood in necrotic areas
Fibrosis	A matrix that seems to extend between the cells of the liver and contains cytoplasm-rich fibroblasts within the matrix and some mononuclear cell infiltrates
Vacuolation	Hepatocytes or areas of the liver, including large and small clear circular vacuoles
Infiltration	All the mononuclear cell infiltrates; that is, both histiocytes and lymphocytes are labeled together.
Portal	Areas including small bile ducts, arteries, and veins
Connective tissue	Large blood vessels, ligaments, and muscle tissue that exist inside or outside the liver tissue.
